# The Diagnosis and Management of Pediatric Blunt Abdominal Trauma—A Comprehensive Review

**DOI:** 10.3390/diagnostics14202257

**Published:** 2024-10-10

**Authors:** Marko Bašković, Dorotea Keretić, Matej Lacković, Marta Borić Krakar, Zenon Pogorelić

**Affiliations:** 1 Department of Pediatric Surgery, Children’s Hospital Zagreb, Ulica Vjekoslava Klaića 16, 10000 Zagreb, Croatia; marko.baskovic@kdb.hr (M.B.);; 2 School of Medicine, University of Zagreb, Šalata 3, 10000 Zagreb, Croatia; 3 Scientific Centre of Excellence for Reproductive and Regenerative Medicine, School of Medicine, University of Zagreb, Šalata 3, 10000 Zagreb, Croatia; 4 Department of Pediatric Surgery, University Hospital of Split, Spinčićeva ulica 1, 21000 Split, Croatia; 5 Department of Surgery, School of Medicine, University of Split, Šoltanska ulica 2a, 21000 Split, Croatia

**Keywords:** blunt abdominal trauma, blunt abdominal injury, spleen injury, liver injury, pancreatic injury, hollow viscus injuries, urinary tract injuries, children, pediatric surgery

## Abstract

Blunt abdominal trauma in childhood has always been full of diagnostic and therapeutic challenges that have tested the clinical and radiological skills of pediatric surgeons and radiologists. Despite the guidelines and the studies carried out so far, to this day, there is no absolute consensus on certain points of view. Around the world, a paradigm shift towards non-operative treatment of hemodynamically stable children, with low complication rates, is noticeable. Children with blunt abdominal trauma require a standardized methodology to provide the best possible care with the best possible outcomes. This comprehensive review systematizes knowledge about all aspects of caring for children with blunt abdominal trauma, from pre-hospital to post-hospital care.

## 1. Introduction

Trauma has always been one of the leading causes of morbidity and mortality in children. Considering the mechanism of occurrence, it is divided into intentional and unintentional injuries [1,2]. The first pediatric trauma centers began to appear in the 1970s and 1980s of the last centuries, shortly after adult trauma centers. Although practices and ideas between adult and pediatric centers were usually complementary, divergences emerged in the management of blunt abdominal trauma. In the aforementioned years, non-operative management of injuries in children was advocated, which was met with resistance from adult-trauma surgeons [3,4,5]. Despite the gradual development of pediatric trauma centers around the world, more than 95% of deaths caused by trauma are still observed in the group of low- and middle-income countries, but even in high-income countries, mortality caused by trauma sometimes reaches a high 40%, which continues to highlight the importance of further education, research, and injury prevention. Considering the specifics, each country should assess its own needs and develop the pediatric trauma care system daily in order to reduce the percentages as much as possible [6,7]. By 2030, traffic accidents will have been estimated to be the fifth leading cause of death worldwide and the seventh leading cause of disability-adjusted life years (DALYs) lost in children [1,6].

Although limb fractures and head injuries are the most common, about 10% of childhood injury deaths are caused by abdominal injuries, either penetrating or blunt, with road traffic accidents as the leading cause [8,9,10,11]. Injuries caused by traffic accidents in children more often result in abdominal injuries because they often use a seat belt [12]. It should also be noted that very common injuries when a child falls from a bicycle are caused by the bicycle handlebar [13]. Abdominal injuries are most often seen in age groups between 5 and 9 years, twice as often in boys, especially in societies with a lower socioeconomic status [1,14,15,16].

The prevention of injuries in children is reflected in three levels, primary (pre-event), secondary (event), and tertiary (post-event). While primary and secondary prevention relates to preventing the occurrence of injuries and minimizing the damage caused as a result of the injury, tertiary prevention is focused on the treatment and rehabilitation of injured children within the framework of pediatric trauma centers [17,18]. Although the World Health Organization (WHO) places the main focus on primary and secondary prevention, in this comprehensive review, we will focus on the steps aimed at the diagnosis and treatment of pediatric blunt abdominal trauma within the framework of modern medicine and current knowledge.

## 2. Prehospital Trauma Care

Before treating an injured child in a specialized pediatric trauma center, initial stabilization and care are extremely important in reducing morbidity and mortality. To this day, the concept of the “golden hour” has been retained as the most significant for reducing mortality and morbidity, both in adults and in children [19,20,21]. Upon the arrival of the emergency medical team at the scene of the injured child, it is necessary to take steps laid out in structured guidelines, from primary survey to stabilization and eventual resuscitation, based on the Advanced Trauma Life Support (ATLS) protocol [22]. The concept of the trimodal distribution of death states that the first peak of death occurs immediately after injury, and only prevention can affect this mortality, the second peak occurs minutes to hours after injury where rapid assessment and treatment (usually within the “golden hour”) reduces mortality, and the third peak occurs in the days or weeks after injury, usually due to infection and multi-organ system failure, and highly sophisticated care in tertiary centers mitigates this delayed mortality [23].

### Primary Survey

Initial assessment and treatment are called the “primary survey” and include *A*—airway maintenance with cervical spine (C-spine) protection, *B*—breathing and ventilation, *C*—circulation with hemorrhage control, *D*—disability (evaluation of neurologic status), and *E*—exposure (complete visualization)/environmental control (the prevention of hypothermia) [22]. In a small child, bleeding control by direct pressure, dressings, or tourniquets takes precedence over everything else, because rapid bleeding can occur in cases of catastrophic external bleeding. Tranexamic acid, if available, should be given in the dose of 15 mg/kg (maximum dose 1 g) [24]. In the context of trauma, the patency and stability of the airway must be quickly assessed, with a simultaneous assessment of the cervical spine. Cervical spine injury is suspected in cases of face and neck injuries, heavy falls, and high-speed traffic accidents. In the mentioned cases, before undertaking advanced interventions on the respiratory tract, it is necessary to immobilize the cervical spine [25]. Regarding breathing, attention should be paid to asymmetric chest movements, the speed and depth of respirations, and auscultatory indicators. If it is determined that the child has pneumothorax, hemothorax, etc., it is necessary to react quickly in the form of intercostal drainage with an intercostal tube or needle decompression with adequate oxygenation [26]. In children with trauma, hypovolemia due to blood loss is the most common cause of shock. The primary assessment of circulation includes the estimation of pulse rate, pulse volume, heart rate and rhythm, and peripheral perfusion like color, temperature and capillary return, and blood pressure. Due to the physiological reserve in children, blood pressure can be maintained despite the loss of up to 45% of circulating blood volume, and tachycardia is usually the first sign of hypovolemia in children. Vascular access should be established with two intravenous cannulas and, if necessary, central venous cannulas including jugular and femoral veins, and even intraosseous access. Volume replacement should be started with crystalloid bolus (20 mL/kg), if necessary, with vasoactive and inotropic drugs. Cardiac arrest requires cardiopulmonary resuscitation [27,28]. Regarding the neurological status, it is necessary to assess the state of consciousness using the Glasgow Coma Scale (GCS). Patients with significant intracranial injuries or increased intracranial pressure (ICP) require treatment in tertiary centers to reduce brain injury from hypoxia, ischemia, and cerebral edema [26,29]. Finally, a complete assessment of the injured child requires undressing, preferably in a warmer environment, usually in an ambulance. Temperature maintenance is usually performed using warmed intravenous fluids, warm and humidified inhaled oxygen, and covering the patient with warm blankets. Well-equipped ambulances and trained personnel within the primary survey can additionally perform basic blood tests, screening radiographs, and focused assessment with sonography in trauma (FAST). Most of the additional examinations usually cannot be performed in an ambulance and should be planned after the child arrives at the hospital as part of the secondary survey [26,30].

## 3. Approach to a Child with Abdominal Trauma

Trauma is the leading cause of death in the pediatric population, with blunt abdominal trauma being the main cause of morbidity, mortality, and permanent disability [31]. Mortality in children is directly related to the number and type of injured structures. Mortality is less than 20% in cases of isolated injury to the liver, spleen, kidney, or pancreas, it increases to 20% if the gastrointestinal tract is involved, and it increases to 50% if major vessels are injured [32]. The abdominal wall of children has a thinner layer of fat and connective tissue and less-developed muscles, providing less protection to the abdominal organs. Due to their increased flexibility, the ribs are less prone to fracture, but they more easily transmit energy to the internal organs, especially the liver and spleen [33,34,35].

After the previously described primary survey, usually upon the child’s arrival at the hospital, the patient undergoes a systematic secondary survey (from head to toe) to determine all of the injuries that were not identified in the primary survey [31]. As part of the secondary survey, the abdomen is thoroughly inspected for visible abrasions, ecchymoses, seat belt sign, and possible distension. The location of the pain is categorized as local or diffuse. Local pain is usually, though not always, associated with minor injuries, while diffuse pain is associated with major injuries. Symptoms of generalized peritonitis (rigidity with rebound tenderness) usually require urgent laparotomy. As a sign of peritoneal irritation, it is important to pay attention to the Kehr sign (pain in the left shoulder caused by palpation of the left upper quadrant). The presence or absence of peristalsis can be determined by auscultation. Absent bowel sounds can be a sign of peritonitis. If possible, it is necessary and important to perform both a rectal examination and an examination of the genitals for the presence of blood [36,37,38,39].

The primary goal in a child with intra-abdominal bleeding is to stabilize it. If there is no possibility of blood transfusion (20 mL/kg), it is necessary to prescribe a crystalloid bolus (up to 3 × 20 mL/kg, up to a maximum of 3 L). For losses > 40%, blood transfusion should be the first choice without further delay. If stabilization does not occur despite the compensation, it is necessary to perform an urgent laparotomy [25,27]. Colloid solutions that include 5% albumin and hydroxyethyl starch (HES) may be used for resuscitation, but there are concerns of the exacerbation of coagulopathy because of the use of HES [40,41]. After the patient has been assessed, resuscitated, and stabilized, observation, laboratory evaluation, imaging, and, if necessary, surgical treatment are carried out within the previously described secondary survey [37,38]. To reduce the risk of aspiration of gastric contents, a nasogastric tube or, in patients with maxillofacial trauma, an orogastric tube should be placed in patients with vomiting or abdominal distension [42].

### 3.1. Laboratory Evaluation

During the diagnostic treatment of a child with blunt abdominal trauma, it is necessary to perform a complete blood count (CBC), blood type and crossmatch, arterial or venous blood gas, alanine aminotransferase (ALT), aspartate aminotransferase (AST), amylase, lipase, serum electrolyte, creatinine, blood urea nitrogen, blood glucose, coagulation, and urinalysis evaluation [43,44]. In hemodynamically unstable patients who do not respond to fluid resuscitation, CBC and blood type and crossmatch should be performed in a way that does not delay surgery, while in hemodynamically stable patients with signs of intra-abdominal injury (IAI), a CT scan of the abdomen and pelvis should be performed immediately without waiting for laboratory findings [31]. In hemodynamically stable patients without signs of IAI, the presence of unexplained anemia, hematuria (≥50 red blood cells per high-powered field), or an increase in AST (>200 international unit/L) or ALT (>125 international unit/L) indicates the need for a CT scan of the abdomen and pelvis [31,45,46]. In hemodynamically unstable patients, hemoglobin and hematocrit should be measured frequently, while in hemodynamically stable patients with suspected IAI, hemoglobin and hematocrit should be monitored every four-to-six hours. Even with serious intra-abdominal injuries with acute blood loss, the initial hemoglobin and hematocrit may be normal, while an initial hematocrit of less than 30% indicates severe blood loss [47]. Until blood type and crossmatch results are available, a patient with life-threatening bleeding should be given 0-negative uncrossmatched packed red blood cells (pRBCs) immediately (initial volume 10 to 20 mL/kg up to 2 units) if crystalloid fluid resuscitation (boluses up to 60 mL/kg, up to a maximum of 3 L) does not reverse hypovolemic shock [48]. Regarding urinalysis, a CT scan of the abdomen and pelvis with intravenous contrast is indicated only in children with gross hematuria and microhematuria with ≥50 RBCs per high-powered field [49]. The elevation of liver transaminases (AST > 200 IU/L or ALT > 125 IU/L) in hemodynamically stable children with blunt abdominal trauma appears to be a sensitive and specific indicator of IAI, and CT of the abdomen and pelvis is indicated in these cases [31,50]. As for serum amylase and lipase levels in children with significant blunt abdominal trauma, they may or may not indicate pancreatic injury, and we only measure them as a basis for potential comparison if pain symptoms persist after the initial assessment [51,52,53]. Patients with coagulopathy warrant fresh frozen plasma (FFP) even in the absence of blood transfusion requirements. Hemostatic resuscitation using blood component therapy resembling that of whole blood (pRBC:FFP:platelets in a 1:1:1 ratio) has been associated with improved outcomes [54].

### 3.2. Imaging

After the clinical examination, radiological diagnostics take central place in the complete assessment of children with blunt abdominal trauma. The leading place for the evaluation of intra-abdominal injuries is occupied by ultrasound, FAST, contrast-enhanced ultrasound (CEUS), and CT of the abdomen and pelvis, with an indication that CT is still the “golden standard” in the diagnosis of pediatric blunt abdominal trauma [55,56].

Plain radiographs have a very limited role in the diagnosis of blunt abdominal trauma [42]. Plain radiography can detect pneumoperitoneum, which is a sign of gastrointestinal perforation. Also, fractures of the ribs and pelvis can arouse suspicion and be an indirect indicator of injury to the abdominal organs, and the central displacement of the bowel loops may indicate free fluid in the abdomen [33,57].

The use of ultrasound in the diagnosis of blunt abdominal trauma is limited but extremely useful in the initial assessment. Ultrasound shows low sensitivity for the direct detection of visceral injuries [55]. The techniques and methods used in the diagnosis of blunt abdominal trauma are FAST and the increasingly popular CEUS, which equates to CT in terms of sensitivity and specificity [58,59]. FAST is a rapid ultrasound examination at the patient’s bedside as a blood-screening test around the heart (pericardial effusion) or abdominal organs (hemoperitoneum). Extended FAST (eFAST) includes additional ultrasound examinations to evaluate pneumothorax. The primary advantage of FAST is primarily the examination of a hemodynamically unstable patient who requires immediate surgery instead of CT. In a hemodynamically stable patient, due to its low sensitivity, it should be interpreted within the clinical status of the patient [60]. A negative FAST essentially implies the absence of hemoperitoneum, not the absence of intra-abdominal injury [61]. CEUS has been shown to have many advantages over routine ultrasound for the detection of abdominal injuries, primarily in higher sensitivity approaching that of CT [62,63,64]. The main problem is that, with CEUS, we cannot consistently identify injuries to smaller organs such as the adrenal gland, especially in a multi-trauma setting [65]. CEUS can avoid the use of ionizing radiation and is particularly useful in monitoring previously known injuries in patients treated nonoperatively [66].

Abdominal and pelvic CT with intravenous (IV) contrast (in a bolus dose of 2 mL/kg) is the gold standard in the diagnosis of blunt abdominal trauma in hemodynamic stable children [67]. Indications for the use of CT are signs of injuries to the abdominal wall (e.g., seat belt sign), abdominal pain or tenderness, the impossibility of examining a very young child, and, in a child with an intellectual disability, positive FAST, initial serum AST > 200 IU/L or ALT > 125 IU/L, initial elevated serum pancreatic enzymes, gross hematuria or microscopic hematuria with ≥50 RBCs per high-powered field, declining or unexplained hematocrit <30%, and thoracic wall trauma with abnormal chest radiograph or absent or decreased breath sounds or hypoxemia [38,68,69]. Considering the increased radiosensitivity in children, it is important to adhere to the indications to maximally rationalize the use of CT, following the ALARA (As Low As Reasonably Achievable) principles [70].

## 4. Spleen Injury

**Figure 1:**
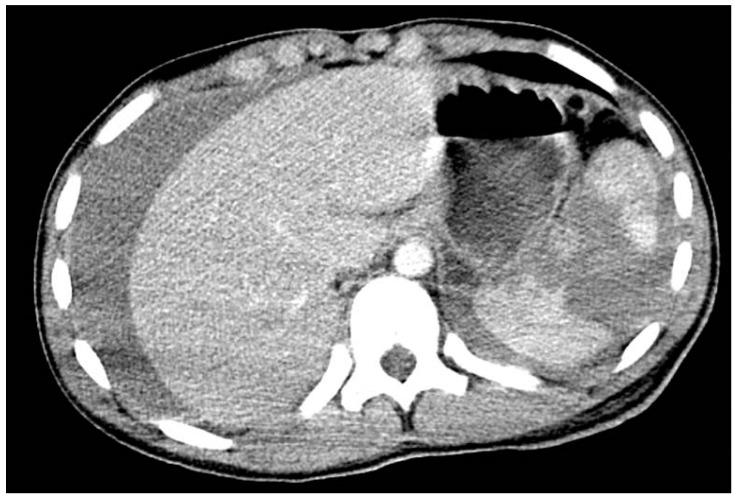
CT with IV contrast shows free fluid around the liver and spleen (hemoperitoneum) with complete disruption of the spleen parenchyma in terms of rupture.

The spleen, in addition to being the most vascularized organ in the body, is the organ most often affected by trauma [71]. In a child with hemodynamic shock, FAST is the only test performed before emergency surgical treatment. Spleen trauma can already be suspected during the initial ultrasound examination based on its heterogeneous appearance with a loss of visualization of its hilum. CEUS can detect injuries that are not visible through a conventional ultrasound, but CT with IV contrast is the gold standard in diagnosis in hemodynamically stable patients, with almost 100% sensitivity and specificity (Figure 1). Lesions are best visible in the venous phase and are usually represented by lacerations. CEUS is extremely useful in monitoring non-operative treated patients [72].

Around the world, there is a paradigm shift in the treatment of blunt splenic injury in hemodynamically stable children towards non-operative treatment. Delayed rupture of the spleen in children is rare [73]. Today, almost 95% of children can be treated non-operatively (grades I–IV, and sometimes even grade V) [74] (Table 1). Complications of nonoperative treatment are rare and include delayed bleeding, abscesses, pseudocysts, and pseudoaneurysms [75,76].

In patients undergoing surgical intervention, an attempt should be made to save the spleen by splenorrhaphy or partial splenectomy (leaving at least one-third of the spleen with an intact arterial blood supply). In cases of other significant intra-abdominal injuries, hemodynamically unstable patients, and an irreparably damaged spleen, splenectomy is the best option [77]. Embolization may help prevent splenectomy, and it is an alternative to surgery for selected hemodynamically stable patients [78,79]. To prevent overwhelming post-splenectomy infection (OPSI), patients should undergo vaccination against encapsulated bacteria including *Streptococcus pneumoniae*, *Haemophilus influenzae type b* (Hib), and *Neisseria meningitidis* [80,81]. Prophylactic antibiotics are usually aimed at the high-risk period (1 to 3 years after splenectomy), children ≤5 years of age, or concomitant immunocompromised patients [82].

## 5. Liver Injury

**Figure 2:**
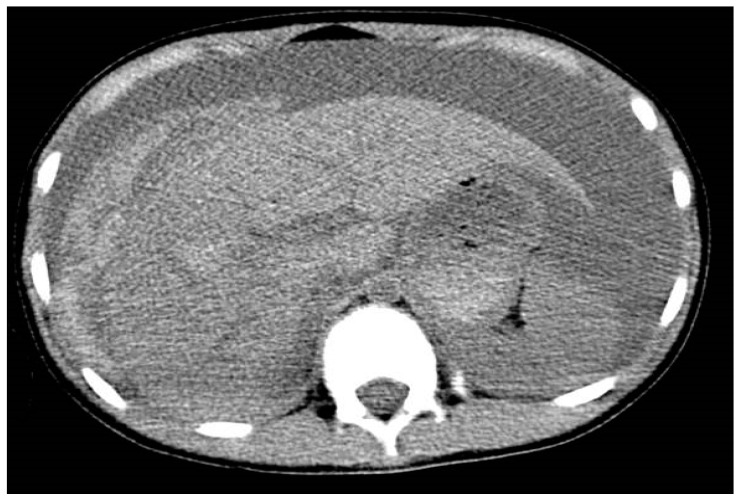
CT shows hematomas within the liver parenchyma, with rupture of the capsule and development of hemoperitoneum.

After spleen injury, blunt liver injury is the second most common injury, but it is much more dangerous with a higher mortality rate. The gold standard in diagnostics for stable patients is CT with contrast (Figure 2, Table 2), while ultrasound (especially CEUS) is ideal for follow-up. It is important to note that the severity of injury assessed by CT does not necessarily correlate with clinical survey and the need for emergency surgery [83]. Initially, FAST can be used in addition to history, physical examination, and laboratory findings to help determine whether further evaluation is needed [84,85].

Nonoperative management of liver injury in children has become the standard of care. Although the majority of injured children can be treated non-operatively, it is still a challenge to recognize a severely injured child who needs surgical intervention in time [86,87]. The indications for emergency surgery following hepatic injury in children include unstable hemodynamic parameters, evidence of continuing bleeding, and the presence of other associated injuries. A small proportion of children who are initially stable will continue to bleed. Usually, patients with early delayed bleeding (<48 h after injury) represent an initial failure to detect continued bleeding. Simple methods of hemostasis like manual compression, hepatorrhaphy, suture ligation of bleeding vessels, omental patches, electrocautery, and topical hemostatic agents can manage most hepatic injuries necessitating surgical intervention [88,89]. Severe bleeding can initially be controlled by compression of the liver itself, compression of the aorta to the spine, or occlusion of the portal triad via the Pringle maneuver [83]. Recently, in centers with available resources, angiographic embolization is increasingly being considered for grade 3 or greater liver injury [90]. The greatest challenge is presented by patients who do not respond to initial treatment with the consequent development of hypothermia (core temperature < 35 °C), coagulopathy (prothrombin time > 16 s), and acidosis (pH < 7.2) when complications begin to complement each other [91]. In these situations, “damage control surgery” is indicated, with the approach of perihepatic packing for hemostasis, resuscitation in the intensive care unit (ICU) setting (patients are rewarmed, oxygen delivery is optimized, and coagulation factors are replaced), and re-exploration with definite surgical management (usually 24 to 72 h after the trauma) [92,93,94]. The packs aim to compress the parenchyma with care to avoid critical compression of the inferior vena cava. The fascia should be left open with the use of temporary abdominal closure techniques (Vacuum-Assisted Closure^®^ appliances, Silastic^®^ sheeting, or surgical towels). For definitive management, treatment of deep parenchymal fractures with compression, followed by ligation of bleeding vessels while avoiding deep sutures of the liver, is recommended. Major hepatic resection is undertaken only when the area to be resected is very severely traumatized or already sequestrated and devoid of blood supply [95,96,97]. Vascular or biliary complications are present in about 10% of cases (hemobilia, arteriovenous fistula, pseudoaneurysm, portal vein thrombosis, portal vein stenosis, fistulas, bilhemia, biloma, bilioperitoneum, and stenosis of the biliary tract). Most of these can be easily managed by conservative and interventional imaging and endoscopic techniques [83,98,99,100].

## 6. Pancreatic Injury

**Figure 3:**
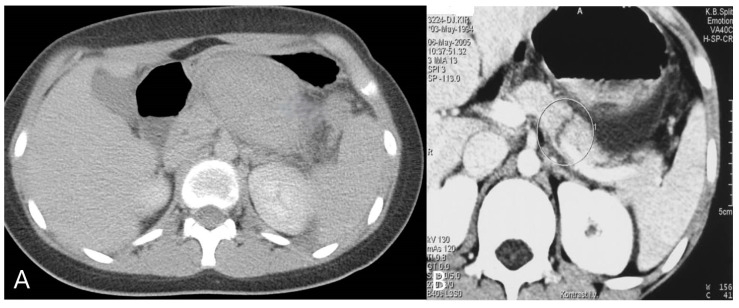
Pancreatic injury: (**A**) Post-contrast CT sections through the abdomen in the region of the omental bursa show a fluid collection corresponding to a hematoma of the omental bursa. (**B**) CT representation of pancreatic rupture.

The retroperitoneal position of the pancreas usually results in a characteristically late presentation of symptoms, and in relation to solid organ injuries, the morbidity associated with pancreatic injury is the highest [101]. CT with IV contrast is the gold standard in the diagnosis of blunt pancreatic injury (Figure 3, Table 3), while endoscopic retrograde cholangiopancreatography (ERCP) and/or magnetic resonance cholangiopancreatography (MRCP) can provide additional information to assess the pancreatic duct and its integrity [102,103]. Ultrasound (especially CEUS) and MRI have been shown to be useful in the follow-up of these patients [71,104,105].

**Figure 4:**
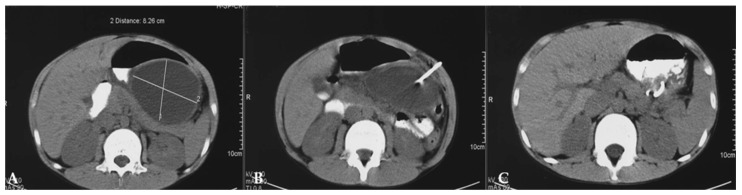
External drainage of huge pancreatic pseudocyst: (**A**) Pseudocyst prior to drainage (8.3 × 6.1 cm); (**B**) CT-assisted extranal percutaneous drainage; (**C**) Complete resolution of the cyst after six weeks.

The generally accepted position is that grade I and II pancreatic injuries are treated non-operatively, including restriction of oral intake initially, intravenous hydration, and parenteral nutrition [106,107]. Although non-operative treatment prolongs hospitalization, time to oral feeding, and full recovery, to this day, the treatment of grade III and higher injuries remains controversial [108]. On the one hand, a more aggressive surgical approach is advocated, while on the other, a more conservative one is advocated with the consequent management of pseudocysts. For grade III, some surgeons will decide on distal pancreatectomy, while others will prefer non-operative treatment. In the case of proximal pancreatic injuries (grade IV and V), some surgeons will prefer significant pancreatic resection or pancreatoduodenectomy, while others will advocate for nonoperative treatment with or without endoscopic stent placement [109,110,111]. Fluid accumulation develops in up to half of children with ductal damage who are initially treated nonoperatively. Some of them develop symptomatic pseudocysts, while other cysts disappear spontaneously [112]. Pseudocysts up to 5 cm in diameter may be treated conservatively, while the large symptomatic pseudocysts can be treated by external or internal drainage (Figure 4) (open or endoscopic drainage into the stomach or bowel, mostly jejunum) [113,114,115,116].

## 7. Hollow Viscus Injuries

Although they are rare, in the context of hollow viscus blunt abdominal trauma, injuries to the small intestine, especially the jejunum (particularly near the ligament of Treitz) or ileum, are the most common, followed by injuries to the duodenum, colon, and stomach. They present themselves subtly, and surgery is almost always required. Timely and accurate diagnosis is the most important for a reduced rate of complications, that is, better patient outcomes [117,118]. Although rapid and affordable, the plain abdominal X-ray showing free gas (pneumoperitoneum) remains the most consistent indicator of bowel injury, but with the indication that the absence of pneumoperitoneum is not a sign of absence of bowel injury. The gold standard in the diagnosis of hollow viscus blunt abdominal injury is CT with IV contrast, which can detect free fluid, bowel-wall thickening or enhancement, hematomas, tearing of the bowel wall, extraluminal air, mesenteric stranding, or extravasation of contrast [71,119,120,121]. Multicenter studies have clearly shown that there is no difference in sensitivity in the identification of intra-abdominal injuries using oral versus intravenous contrast [122]. Diagnostic laparoscopy can be important in the evaluation of children with suspected gastrointestinal perforation due to blunt abdominal trauma. At the same time, in the hands of a skilled surgeon, laparoscopy can be both a diagnostic and therapeutic method [123,124,125]. Antibiotics (for Gram-negative and anaerobic organisms) should only be administered once the diagnosis of hollow viscus injury is confirmed clinically or radiographically to avoid masking signs and symptoms of peritonitis and delaying surgical intervention [126].

### 7.1. Small Intestine Injury

Injuries to the small intestine usually occur in the area of the Treitz ligament and the ileocecal junction, that is, in the area of relative fixations at the antimesenteric border [127]. When the small intestine is injured, it is necessary to assess whether it is a minor injury, where the tissue is not devitalized and primary repair in one or two layers is possible, or a major injury (if the injury involves more than 50% the circumference, grade III, IV, V), which often requires segmental bowel resection and anastomosis formation (Table 4). During resection and reconstruction, care must be taken to not narrow the intestinal lumen (tension-free repair by suturing in a transverse manner), to prevent possible strictures. Repair with hand-sewn or stapled anastomosis has been shown to have similar complications [117,118,128,129].

### 7.2. Duodenum Injury

Low-grade duodenal injuries (grade I, II) usually present as progressive obstruction of the gastric outlet 48-72 h after the injury. Duodenal hematomas with gastric decompression and total parenteral nutrition usually disappear spontaneously within 1–3 weeks. Taking care not to narrow the lumen, low-grade injuries can be resolved with primary repair after debridement of devitalized tissue in a transverse manner in two layers [130,131]. High-grade injuries (grade III, IV, V) represent a challenge (Table 5). After the duodenum is mobilized (Kocher maneuver), treatment options are end-to-end duodenostomy, tension-free Roux-en-Y duodenojejunostomy, or Billroth II (antrectomy and gastrojejunostomy). For unstable patients, placement of a duodenostomy tube within the defect provides a “damage control” option. Combined duodenal and pancreatic head injuries may require pancreaticoduodenectomy (Whipple’s procedure) [132,133,134].

### 7.3. Colon and Rectum Injury

Recently, the position is that timely (within 6 h) primary repair is the method of choice for grade I-III colon injuries [135]. If the procedure is performed on time, segmental resection and primary anastomosis is the method of choice for high-grade injuries (grade IV, V), while in patients with multiple injuries, hemodynamic instability, comorbidities, and untimely access, the method of choice is resection and colostomy formation (Table 6) [136,137]. Regarding intraperitoneal rectal injuries, the attitudes are the same as for colon injuries, while for extraperitoneal rectal injuries, an opinion was formed that for non-destructive injuries, the feasibility of transanal primary repair should be evaluated. For destructive injuries (grade IV, V), the method of choice is resection and colostomy formation (Table 7) [138,139,140,141].

### 7.4. Stomach Injury

Since the chest partially encloses the stomach, stomach injuries are the rarest. Still, even when serious injuries in the form of perforation occur, they are usually easily diagnosed because they quickly present with peritonitis and a large amount of free air, unlike intestinal injuries. Rapid surgical intervention is important because it significantly reduces mortality [142]. Grade I-III injuries are usually amenable to primary repair with a double-layer suture. Grade IV and V injuries are usually associated with injuries to other organs and usually require subtotal or less often total gastrectomy, while gastroduodenostomy (Billroth I), gastrojejunostomy (Billroth II), or Roux-en-Y reconstruction is used to restore gastrointestinal continuity (Table 8) [143,144,145].

## 8. Urinary Tract Injuries

### 8.1. Kidney Injury

**Figure 5:**
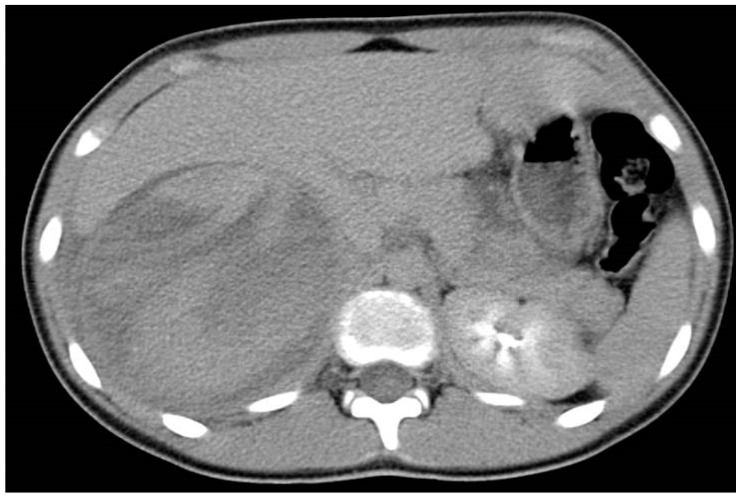
CT with intravenous contrast shows a laceration of the right kidney with perirenal hematoma.

Due to its size, reduced perirenal fat, and weaker abdominal musculature, the kidney is the most frequently injured urinary tract organ in children [146]. Hematuria is an important sign of kidney damage even in microscopic form, but the absence of hematuria does not rule out kidney and urinary tract involvement [147]. CT with IV contrast is the radiological examination of choice in the evaluation of kidney injuries in hemodynamically stable patients (Figure 5, Table 9). Kidney imaging is indicated for children with blunt injuries in the presence of gross hematuria or microscopic hematuria (≥3 to 5 RBCs/HPF). Relative indications for imaging include a significant decelerating mechanism, such as a high-speed motor vehicle collision or a fall from height, or clinical features suggestive of kidney injury, including flank bruising or tenderness [148]. If there are no indications for CT, CEUS is the method of choice, but this technique cannot detect injuries of the urinary tract or collecting system because the contrast medium is not excreted by the kidneys. CEUS is important in follow-up [149,150]. Nonoperative treatment with clinical and radiological follow-up has become the standard approach in the treatment of blunt renal trauma in children. Patients with urinary leakage have greater morbidity, including febrile episodes and an increased need for operative or image-guided interventions. Emergency intervention is indicated only for hemodynamic instability caused by bleeding, and, if available, angioembolization has an advantage over open surgical intervention [151,152,153]. Grade V injuries sometimes require partial or total nephrectomy, while nonviable renal tissue is not an indication for surgery [154,155]. Complications include arteriovenous fistula and pseudoaneurysm associated with injuries to the renal vessels, which can be effectively treated with embolization. Most small uninfected urinomas regress spontaneously, while large ones require percutaneous drainage or endoscopic placement of ureteral stents [156,157].

### 8.2. Ureter Injury

Ureteral injuries are more often caused by penetrating than blunt trauma, and the diagnostic accuracy of CT is best during a delayed CT scan up to ten minutes after contrast injection (Table 10). The most sensitive radiological diagnostic test is the retrograde pyelogram [158]. Often, ureteral injuries are missed during initial evaluation because signs and symptoms are minimal and nonspecific. Primary repair of partial lacerations should be followed by internal stenting. The management of complete lacerations, avulsions, or crush injuries depends on the amount of ureter lost and its location [159]. If there is an adequate healthy length of the ureter, a primary ureteroureterostomy can be performed. If primary ureteroureterostomy is not achievable, distal ureteral injuries can be managed using a psoas bladder hitch, Boari flap, or nephropexy. Proximal injuries can be managed using transureteroureterostomy, auto-transplantation, or ureteral replacement with the bowel or appendix [160,161,162].

### 8.3. Bladder Injury

Blunt bladder injuries are usually associated with blunt trauma to the lower abdomen (usually when the bladder is distended with urine—intraperitoneal) or pelvic fractures (extraperitoneal) [163]. The most characteristic signs of bladder injury are suprapubic pain, the inability to urinate, and hematuria. Ultrasound examination of the bladder can detect free abdominal fluid in the case of intraperitoneal bladder injury, but ultrasound cannot distinguish urine from blood. The optimal diagnostic when a bladder injury is suspected is a retrograde cystography or a retrograde CT cystography with the bladder filled (Table 11) [164,165]. Contusions are treated with catheter drainage. Intraperitoneal ruptures are treated by surgical exploration and primary repair (in two layers with absorbable sutures), with post-operative drainage with a suprapubic tube or transurethral catheter drainage, depending on the possibilities. Extraperitoneal ruptures are treated non-operatively, with transurethral catheter drainage for seven to ten days, unless there are bone fragments inside the bladder that must be removed [166,167,168,169].

## 9. Hospital Discharge and Post-Discharge Care

Although the length of hospital stay (LOS) has been promoted for years as a “grade plus one day”, current APSA (American Pediatric Surgical Association) guidelines recommend hospital discharge based on clinical status rather than the grade of injury, and the patient is considered fit for discharge if vital signs have normalized and if the patient tolerates a regular diet, with minimal abdominal pain [170,171]. A shift to treatment based on hemodynamic status rather than injury grade has resulted in a significant reduction in LOS without an increase in adverse events [172,173,174]. As for activity restrictions, the thesis of “grade plus two weeks” has been retained [170,171]. Reimaging is not recommended routinely but only selectively from patient to patient, depending on the symptomatology of possible complications, initially verified injuries of grade III or higher. For follow-up, the methods of choice are ultrasound or CEUS and, in selected cases, MR [175,176,177,178].

## 10. Artificial Intelligence

In recent years, the development of artificial intelligence has greatly contributed to diagnostic and management knowledge in the field of blunt abdominal trauma. As the rapid and accurate interpretation of CEUS and CT is still a challenge, detection models based on a deep learning algorithm have been developed that can simultaneously recognize multiple organ injuries with high sensitivity and specificity [179,180,181,182]. In addition to radiological ones, algorithms are being constructed that include both laboratory and physical parameters [183,184]. Modern machine learning relies on a strict experimental methodology consisting of a three-step process. An exploratory analysis of data is used to choose several different computer algorithms. The metric of interest is then determined, each algorithm is optimally fitted to the training data set, and error is measured. Standard statistical methods are used to compare all the fitted models to the baseline rate in the test set to find the model with the greatest predictive power. The best predictive power is given to the algorithm with the most statistically superior outcomes [185]. Artificial intelligence is likely to play an increasing role in the years to come, reducing the amount of time and effort for clinicians while increasing the rate of sensitivity and specificity [186].

## 11. Conclusions

Treatment of blunt abdominal trauma in children is a topic of great interest, with constant reevaluation of guidelines and new studies every day. Diagnostic and treatment strategies, with the development of technologies and artificial intelligence, are rapidly striving to further reduce unnecessary and invasive procedures in hemodynamically stable children, with as few complications as possible and faster and shorter recovery. Future research, especially those with high levels of evidence, should be further directed towards reaching a consensus on all issues of interest so that children receive the most appropriate optimized care.

## Figures and Tables

**Table 1:** AAST classification of splenic injury.

Grade	Description
I	Subcapsular hematoma < 10% surface area Parenchymal laceration < 1 cm depth
II	Subcapsular hematoma 10–50% surface area; intraparenchymal hematoma < 5 cm Parenchymal laceration 1–3 cm
III	Subcapsular hematoma >50% surface area; ruptured subcapsular or intraparenchymal hematoma ≥ 5 cm Parenchymal laceration > 3 cm depth
IV	Any injury in the presence of a splenic vascular injury or active bleeding confined within the splenic capsule Parenchymal laceration involving segmental or hilar vessels producing > 25% devascularization
V	Any injury in the presence of a splenic vascular injury with active bleeding extended beyond the spleen into the peritoneum Shattered spleen

AAST—American Association for the Surgery of Trauma.

**Table 2:** AAST classification of liver injury.

Grade	Description
I	Subcapsular hematoma < 10% surface area Parenchymal laceration < 1 cm depth
II	Subcapsular hematoma 10–50% surface area; intraparenchymal hematoma < 10 cm Laceration 1–3 cm in depth and ≤ 10 cm length
III	Subcapsular hematoma > 50% surface area; ruptured subcapsular or parenchymal hematoma; intraparenchymal hematoma > 10 cm Laceration > 3 cm depth Any injury in the presence of a liver vascular injury or active bleeding contained within liver parenchyma
IV	Parenchymal disruption involving 25–75% of a hepatic lobe Active bleeding extending beyond the liver parenchyma into the peritoneum
V	Parenchymal disruption > 75% of hepatic lobe Juxtahepatic venous injury to include retrohepatic vena cava and central major hepatic veins
VI	Hepatic avulsion

AAST—American Association for the Surgery of Trauma.

**Table 3:** AAST classification of pancreatic injury.

Grade	Description
I	Minor contusion without duct injury Superficial laceration without duct injury
II	Major contusion without duct injury or tissue loss Major laceration without duct injury or tissue loss
III	Distal transection or parenchymal injury with duct injury
IV	Proximal transection or parenchymal injury involving ampulla
V	Massive disruption of pancreatic head

AAST—American Association for the Surgery of Trauma.

**Table 4:** AAST classification of small intestine injury.

Grade	Description
I	Contusion or hematoma without devascularization Partial thickness, no perforation
II	Laceration < 50% of circumference
III	Laceration > 50% of circumference without transection
IV	Transection of the small bowel
V	Transection of the small bowel with segmental tissue loss Devascularized segment

AAST—American Association for the Surgery of Trauma.

**Table 5:** AAST classification of duodenum injury.

Grade	Description
I	Involving single portion of duodenum Partial thickness, no perforation
II	Involving more than one portion Disruption < 50% of circumference
III	Disruption 50–75% of circumference of D2 Disruption 50–100% of circumference of D1, D3, D4
IV	Disruption > 75% of circumference of D2 Involving ampulla or distal common bile duct
V	Massive disruption of duodenopancreatic complex Devascularization of duodenum

AAST—American Association for the Surgery of Trauma. D1—first position of duodenum; D2—second portion of duodenum; D3—third portion of duodenum; and D4—fourth portion of duodenum.

**Table 6:** AAST classification of colon injury.

Grade	Description
I	Contusion or hematoma without devascularization Partial thickness, no perforation
II	Laceration < 50% of circumference
III	Laceration > 50% of circumference without transection
IV	Transection of the colon
V	Transection of the colon with segmental tissue loss Devascularized segment

AAST—American Association for the Surgery of Trauma.

**Table 7:** AAST classification of rectum injury.

Grade	Description
I	Contusion or hematoma without devascularization Partial thickness laceration
II	Laceration < 50% of circumference
III	Laceration > 50% of circumference
IV	Full-thickness laceration with extension into the perineum
V	Devascularized segment

AAST—American Association for the Surgery of Trauma.

**Table 8:** AAST classification of stomach injury.

Grade	Description
I	Contusion/hematoma Partial thickness laceration
II	Laceration < 2 cm in GE junction or pylorus <5 cm in proximal 1/3 stomach <10 cm in distal 2/3 stomach
III	Laceration >2cm in GE junction or pylorus >5 cm in proximal 1/3 stomach >10 cm in distal 2/3 stomach
IV	Tissue loss or devascularization <2/3 stomach
V	Tissue loss or devascularization >2/3 stomach

AAST—American Association for the Surgery of Trauma; GE—gastroesophageal.

**Table 9:** AAST classification of kidney injury.

Grade	Description
I	Subcapsular hematoma and/or parenchymal contusion without laceration
II	Perirenal hematoma confined to Gerota’s fascia Renal parenchymal laceration ≤ 1 cm depth without urinary extravasation
III	Renal parenchymal laceration > 1 cm depth without collecting system rupture or urinary extravasation Any injury in the presence of a kidney vascular injury or active bleeding contained within Gerota’s fascia
IV	Parenchymal laceration extending into urinary collecting system with urinary extravasation Renal pelvis laceration and/or complete ureteropelvic disruption Segmental renal vein or artery injury Active bleeding beyond Gerota’s fascia into the retroperitoneum or peritoneum Segmental or complete kidney infarction(s) due to vessel thrombosis without active bleeding
V	Main renal artery or vein laceration or avulsion of hilum Devascularized kidney with active bleeding Shattered kidney with loss of identifiable parenchymal renal anatomy

AAST—American Association for the Surgery of Trauma.

**Table 10:** AAST classification of ureter injury.

Grade	Description
I	Contusion or hematoma without devascularization
II	<50% transection
III	>50% transection
IV	Complete transection with <2 cm devascularization
V	Avulsion with >2 cm devascularization

AAST—American Association for the Surgery of Trauma.

**Table 11:** AAST classification of bladder injury.

Grade	Description
I	Contusion, intramural hematoma Partial thickness laceration
II	Extraperitoneal bladder wall laceration < 2 cm
III	Extraperitoneal (>2 cm) or intraperitoneal (<2 cm) bladder wall laceration
IV	Intraperitoneal bladder wall laceration > 2 cm
V	Intraperitoneal or extraperitoneal bladder wall laceration extending into the bladder neck or ureteral orifice (trigone)

AAST—American Association for the Surgery of Trauma.

## Data Availability

Not applicable.
